# Nano-Montmorillonite Regulated Crystallization of Hierarchical Strontium Carbonate in a Microbial Mineralization System

**DOI:** 10.3390/ma12091392

**Published:** 2019-04-29

**Authors:** Kui Zheng, Tao Chen, Jian Zhang, Xiuquan Tian, Huilin Ge, Tiantao Qiao, Jia Lei, Xianyan Li, Tao Duan, Wenkun Zhu

**Affiliations:** 1 Analytical and Testing Center, Southwest University of Science and Technology, Mianyang 621010, China; zhengkui@swust.edu.cn; 2 State Key Laboratory of Environmentally Friendly Energy Materials, Southwest University of Science and Technology, Mianyang 621010, China; chentao314710@163.com (T.C.); z2358727542@163.com (J.Z.); tian1993tian1993@163.com (X.T.); gehuilin1998@163.com (H.G.); qtt1805338097@163.com (T.Q.); cutiancheng@163.com (J.L.); lxying@swust.edu.cn (X.L.); duant@ustc.edu.cn (T.D.); 3 Nuclear Waste and Environmental Safety Key Laboratory of Defense, National Collaborative Innovation Center for Nuclear Waste and Environmental Safety, Southwest University of Science and Technology, Mianyang 621010, China

**Keywords:** biomineralization, microorganism, strontium carbonate, nano-montmorillonite

## Abstract

In this paper, nano-montmorillonite (nano-MMT) was introduced into the microbial mineralization system of strontium carbonate (SrCO_3_). By changing the nano-MMT concentration and the mineralization time, the mechanism of mineralization was studied. SrCO_3_ superstructures with complex forms were acquired in the presence of nano-MMT as a crystal growth regulator. At low concentrations of nano-MMT, a cross-shaped SrCO_3_ superstructure was obtained. As the concentration increased, flower-like SrCO_3_ crystals formed via the dissolution and recrystallization processes. An emerging self-assembly process and crystal polymerization mechanism have been proposed by forming complex flower-like SrCO_3_ superstructures in high concentrations of nano-MMT. The above research indicated that unique bionic synthesis strategies in microbial systems could not only provide a useful route for the production of inorganic or inorganic/organic composites with a novel morphology and unique structure but also provide new ideas for the treatment of radionuclides.

## 1. Introduction

Biomineralization refers to the process that an organism constructs a hierarchical structure based on inorganic minerals under certain environmental conditions [1,2,3,4,5]. This process is highly regulated by the biological environments that guide mineral nucleation and growth, including solution states, biomacromolecules, and substrates [6,7,8]. Although the main components of many mineralized tissues are the inorganic phases regulated by the upper biological environment during crystallization and growth. The inorganic–organic advanced hybrid materials formed by the biomineralization process have unmatched physical and chemical properties compared with synthetic materials. Inspired by biomineralization in nature, some artificial mineralizations using bio-excitation techniques have attracted considerable attention.

Strontium carbonate (SrCO_3_) is an important industrial raw material commonly used in the preparation of electronic components, spectroscopic reagents, pyrotechnic materials, rainbow glass and other barium salts. At present, studies on the control of particle size and morphology of SrCO_3_ particles have become a hot topic. Commonly used preparation methods of SrCO_3_ mainly include the supergravity method, solid-phase synthesis method, liquid-phase precipitation method, and microemulsion method [9,10,11,12,13], etc. These methods have successfully prepared SrCO_3_ in the form of needles, spindles, flakes, dumbbells, olives, and other similar morphologies.

Soil mineral–microbial interactions are the most basic biogeochemical system [4,14,15,16,17,18]. As an important clay mineral, montmorillonite (MMT) often interacts with microorganisms at the cellular level, participating in adsorption, aggregation, nucleation and mineralization. Dong et al. [19] used Sheva bacteria to directly act on iron-rich clay minerals to reduce Fe^3+^ into Fe^2+^ in the clay minerals, and used the valence transition of the process to treat variable-valent uranium pollution. Recent advances in the understanding of the role and application of microorganisms for the remediation of toxic metal and radionuclide-contaminated sites have also been reported. These advances include the use of natural bacteria or genetically engineered bacteria to immobilize metal ions and radionuclides using microbial enrichment and mineralization effects [20,21,22,23].

Previous research has concentrated on the use of microbial mineralization to achieve the purpose of mineralizing radionuclides, and few researchers have utilized microbial mineralization techniques to achieve regulation of the SrCO_3_ morphology. Herein, we used carbonate mineralized bacteria (Bacillus pasteurii) as research objects to study the effect of nano-montmorillonite (nano-MMT) on the morphology of SrCO_3_ by changing the amount of nano-MMT and mineralization time. The results show that nano-MMT played a pivotal role in the dissolution and recrystallization of SrCO_3_. In this process, with the increase in the amount of nano-MMT, nano-MMT regulated the transformation of SrCO_3_ from cross-shaped to flower-like. In addition, we have found that the mineralization capacity of microorganisms for Sr^2+^ can reach more than 95% in the presence of nano-MMT. This work not only provides some theoretical references for understanding the biomineralization mechanism but also provides some experiences for microbial mineralization and consolidation of radionuclides.

## 2. Materials and Methods

### 2.1. Materials

Strontium nitrate, peptone, NaCl, glucose, MMT and anhydrous ethanol were purchased from Aladdin (Chengdu, China). All chemicals were not purified prior to use. And water was deionized.

### 2.2. Strain Culture

The liquid mediums were prepared by adding sodium chloride (10 g), glucose (20 g), and peptone (10 g) to 1 L of deionized water (DIW) and the pH was regulated to 8.0 with HCl and NaOH. The Bacillus pasteurii (ATCC 11859) was further inoculated into as-prepared liquid medium and cultured in the thermostatic biochemical incubator at 30 °C for 24 h. The morphology of Bacillus pasteurii is shown in Supporting Information, Figure S1.

### 2.3. Preparation of Nano-MMT Solution

A quantity of 1 g MMT was added to 1 L deionized water and then stirred at 500 r min^−1^ for one week. The above solutions were then centrifuged at 3000 rpm for 3 min and the supernatant was the desired single layer of nano-MMT solution.

### 2.4. Preparation of Strontium Carbonate Nanocrystals by Microbial Mineralization in the Presence of Nano-MMT

The as-prepared liquid mediums were further placed in the high-pressure steam sterilization pot at 120 °C for 20 min, then taken out on the clean bench for natural cooling. Then, 0.03 mol/L urea/Sr(NO_3_)_2_ was further added to the as-prepared liquid medium via the microporous membrane. Next, 0 mL, 3 mL, 6 mL, 9 mL and 12 mL nano-MMT solution were respectively injected into a the as-prepared liquid medium using a pipette. Finally, 3 mL of the concentrated bacterial liquid was injected into the as-prepared liquid medium and then placed in the thermostatic biochemical incubator for 6 h, 12 h, and 18 h, respectively. The obtained strontium carbonate nanocrystals were further washed three times with absolute ethanol and pure water, respectively.

### 2.5. Characterization

The morphology and lattice of strontium carbonate were observed using SEM (Zeiss, Germany) and TEM (Zeiss, Germany), respectively. The functional groups of strontium carbonate were examined using X-ray photoelectron spectroscopy (XPS, Kratos Analytical, Kratos Tech, Manchester, UK) and FT-IR (Thermo Nicolet,Waltham, MA, USA), respectively. The crystalline state of strontium carbonate was examined using XRD (X Pert pro, PANalytical, Almelo, The Netherlands).

## 3. Results and Discussion

### 3.1. Effects of Mineralization Time on the Morphology of Strontium Carbonate in the Absence of Nano-MMT

**Figure 1:**
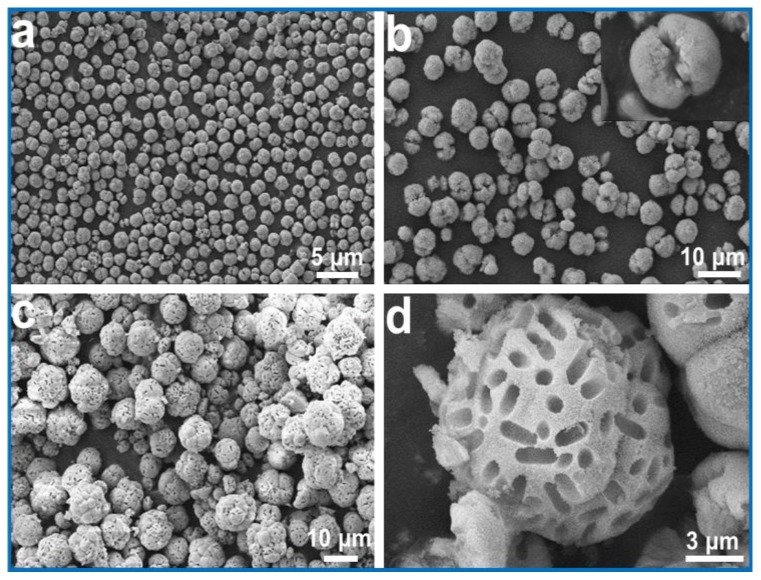
Typical SEM images of SrCO_3_ nanocrystals obtained in the absence of nano-montmorillonite (nano-MMT) when the mineralization time were 6 h (**a**), 12 h (**b**), 18 h (**c**,**d**), respectively.

Crystallization processes of SrCO_3_ nanocrystals were typically observed in the absence of nano-MMT in a range of times. During different periods, different morphologies of SrCO_3_ were obtained through microbial mineralization. Figure 1a shows a representative scanning electron microscopy (SEM) image of SrCO_3_ particles which have a slightly spherical shape, obtained after a mineralization time of 6 h. Figure 1b shows dumbbell-like SrCO_3_ obtained after a mineralization time of 12 h. Interestingly, with the increase of the mineralization time, the dumbbell-like SrCO_3_ turned into popcorn-like SrCO_3_ via the process of dissolution and recrystallization, see Figure 1c. By magnifying Figure 1c, we can clearly see that there are many voids left by the death of the bacteria on the surface of the SrCO_3_ crystal, indicating that the microorganism acts as a nucleation site for SrCO_3_ crystallization, which is consistent with the experimental results of Chen et al. [24,25,26]. The change in the surface morphology could reflect the dissolution and recrystallization process of nano-spherical SrCO_3_.

**Figure 2:**
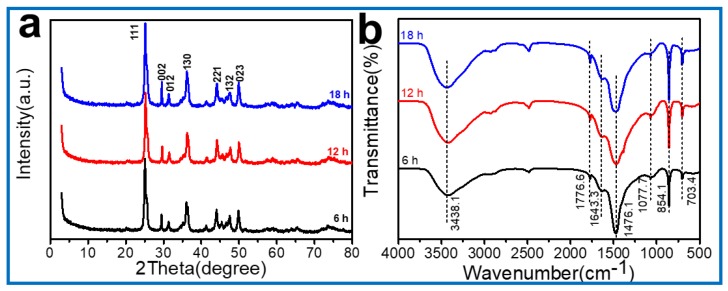
(**a**) XRD patterns and (**b**) Fourier-transform infrared (FT-IR) spectrum of strontium carbonate obtained in the absence of nano-MMT when the mineralization time was 6 h, 12 h, and 18 h, respectively.

The SrCO_3_ crystal collected in the absence of nano-MMT over different periods of time were further examined using X-ray diffraction (XRD). The different diffraction angle position 2θ of 24.9°, 29.3°, 31.5°, 36.1°, 40.9°, 43.9°, 47.9° and 50.1° approximately correspond to (111), (002), (012), (130), (220), (221), (132) and (023) of SrCO_3_ (PDF#05-0418), respectively, indicating that the mineralized samples were orthorhombic SrCO_3_, see Figure 2a [27]. The XRD diffraction peaks are approximately the same under different time conditions, indicating that the crystallized properties of the mineralized SrCO_3_ crystals are superior.

As an effective and convenient means of distinguishing different crystal phases of strontium carbonate, Fourier-transform infrared spectroscopy (FT-IR) was adopted. The typical characteristic peak of 3438.1 cm^−1^ was attributed to the -O-H bond asymmetric stretching vibration and symmetric stretching vibration, which is due to the water present on the surface of the SrCO_3_ crystals, see Figure 2a. The absorption peaks at 703.4/854.1 cm^−1^ were characteristic vibrations of the CO_3_^2−^ out-of-plane and in-plane bending vibration. Absorption bands at 1077.7 and 1476.1 cm^−1^ could be ascribed to the asymmetric and symmetric stretching vibration peaks of CO_3_^2−^. The characteristic peak of the amide group appearing at 1643.3 cm^−1^ indicates that the soluble protein produced by bacterial metabolism participated in the mineralization process of SrCO_3_.

### 3.2. Effects of Mineralization Time on Morphology of Strontium Carbonate in the Presence of Nano-MMT

**Figure 3:**
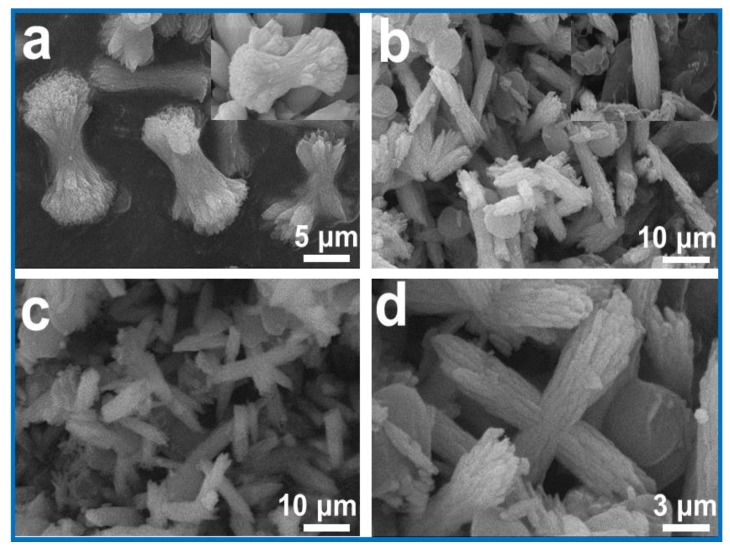
Typical SEM images of SrCO_3_ obtained under a nano-MMT concentration of 2 mL/L when the mineralization times were 6 h (**a**), 12 h (**b**), (**c**,**d**) 18 h, respectively.

The physicochemical characterization of single-layer nano-MMT is shown in Supporting Information, Figure S2. The morphology of the SrCO_3_ could be successfully regulated by the effect of nano-MMT. Figure 3 shows, in detail, the SEM images of SrCO_3_ collected from the microbial mineralization system of nano-MMT as additives under the concentration of nano-MMT is 2 mL/L. A novel bone-like SrCO_3_ crystal was obtained after the mineralization time of 6 h, see Figure 3a. Interestingly, the bone-like SrCO_3_ crystals gradually transformed into rod-like SrCO_3_ crystals when the mineralization time increased to 12 h, see Figure 3b. As the mineralization time increased, the bone-like SrCO_3_ crystals were transformed into cross-shaped SrCO_3_ crystals via dissolution and recrystallization, see Figure 3c. The above results indicate that the single layer nano-MMT has a certain regulation effect on the morphology of SrCO_3_ nanocrystals.

**Figure 4:**
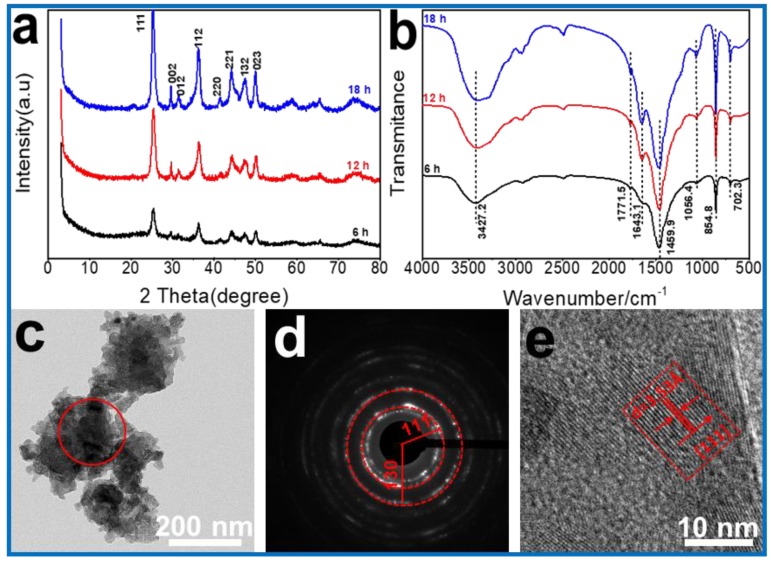
(**a**) XRD patterns and (**b**) FT-IR spectrum of SrCO_3_ obtained over a range of times with a nano-MMT concentration of 2 mL/L, (**c**) TEM image of a SrCO_3_, (**d**) selected area electron diffraction (SAED) pattern shows that this block is polycrystalline SrCO_3_; (**e**) high-resolution transmission electron microscopy (HRTEM) image of the SrCO_3_ showing continuous (111) lattice fringes.

Figure 4a shows that the diffraction peaks of the (111) and (112) crystal faces of the SrCO_3_ gradually increase as the mineralization time increased, indicating that the crystallinity was increased. Furthermore, the analysis results of the FT-IR spectrum are consistent with the XRD pattern, see Figure 4b. High-resolution transmission electron microscopy (HRTEM) and selected area electron diffraction (SAED) were applied to characterize the SrCO_3_ crystal after reaction, see Figure 4c–e. The SAED analysis of a number of nano-SrCO_3_ nanocrystals confirmed the polycrystalline nature, see Figure 4d [28,29]. As shown in Figure 4e, the lattice fringes of SrCO_3_ nanocrystals display interplanar spacings of 0.353 nm in the block, which are consistent with those of the (111) plane of the SrCO_3_ nanocrystals. These observations were further confirmed by the XRD and FT-IR results.

### 3.3. Effects of Nano-MMT Concentrations on Morphology of Strontium Carbonate

**Figure 5:**
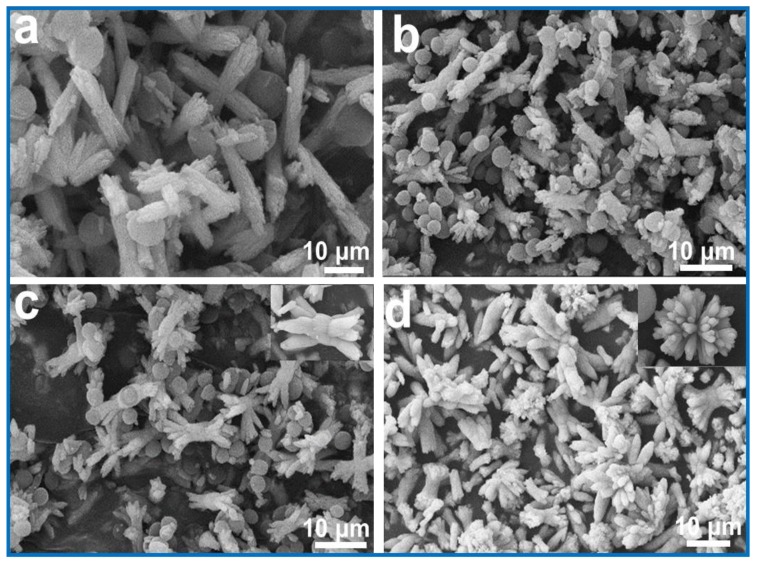
Typical SEM images of SrCO_3_ obtained at the mineralization time of 6 h with a Nano-MMT concentration of 2 mL/L (**a**), 4 mL/L (**b**), 6 mL/L (**c**) and 8 mL/L (**d**), respectively.

To further explore the formation mechanism of cross-shaped SrCO_3_ in the presence of Nano-MMT, varied amounts of nano-MMT were injected into the microbial mineralization system. When the amount of nano-MMT was kept at 4 mL, the SrCO_3_ crystals possessed two morphologies, namely, spherical and flower-like fusiform, see Figure 5b. When the amounts of nano-MMT increased to 6 mL, the flower-like fusiform SrCO_3_ crystals were the predominant form, see Figure 5c. Further, when the amounts of nano-MMT increased to 8 mL, unique flower-like SrCO_3_ crystals were obtained in the microbial mineralization system, see Figure 5d. In the formation process of a flower-like superstructure, rod-like SrCO_3_ crystals aggregated and slowly transformed into flower-like SrCO_3_ crystals via dissolution and recrystallization. The concentration of nano-MMT has a significant influence on the formation of SrCO_3_ crystals with unique morphologies because nano-MMT determined the supersaturation of SrCO_3_ in mineralized systems. The formation of flower-like SrCO_3_ crystals may be attributed to the high concentration of nano-MMT which led to excessive accumulation of rod-like SrCO_3_ crystals around the monolayer nano-MMT [26].

Different amounts of nano-MMT could lead to the formation of varying phases of SrCO_3_ as proven by observation of the XRD patterns, see Supporting Information, Figure S3a. As the amount of nano-MMT increases, the characteristic peaks of (111) and (112) in the XRD pattern are significantly enhanced. In addition, the intensity of the remaining characteristic peaks is relatively increased. The above results indicated that the concentration of nano-MMT has a certain degree of influence on the crystallization of SrCO_3_. Moreover, the results of FT-IR spectrum can further confirm the results of XRD patterns, see Supporting Information, Figure S3b.

**Figure 6:**
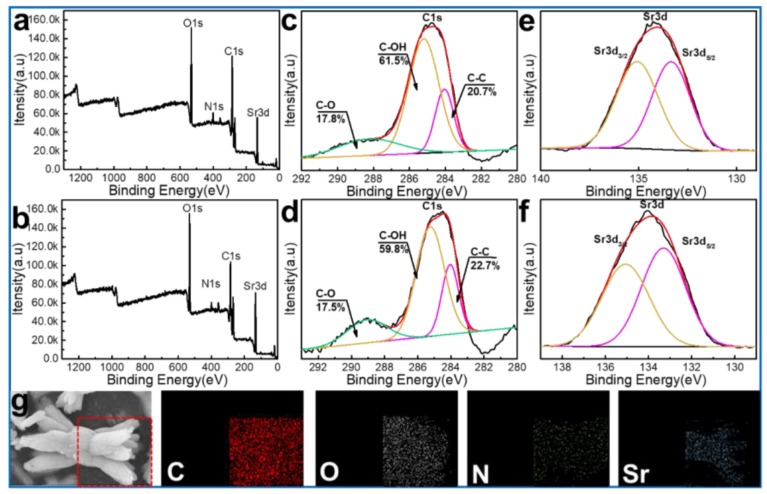
X-ray photoelectron spectroscopy (XPS) spectrum of SrCO_3_ obtained in the absence of nano-MMT (**a**) and the presence of nano-MMT (**b**), respectively. C1s in the absence of nano-MMT (**c**) and the presence of nano-MMT (**d**), respectively. Sr3d in the absence of nano-MMT (**e**) and the presence of nano-MMT (**f**), respectively. (**g**) The EDS element mapping of the SrCO_3_ obtained in the presence of nano-MMT.

X-ray photoelectron spectroscopy (XPS) is used to analyze the chemical functional groups and surface atoms of SrCO_3_. The C1s in SrCO_3_ mainly exist in three chemical states, see Figure 6c,d. Compared with the SrCO_3_ obtained in the absence of nano-MMT, the three chemical states of C1s did not change significantly, indicating that the addition of nano-MMT did not significantly change the crystalline phase of SrCO_3_. The Sr3d spectrum was mainly attributed to the 3d3/2 and 3d5/2 (generated by spin-orbit splitting) at 346.9 and 350.4 eV, respectively, which could be assigned to SrCO_3_, see Figure 6e,f. Energy dispersive spectrometery (EDS) was utilized to further comprehend the composition of various elements in SrCO_3_ crystals. The EDS element mapping showed that the Sr, C, O and N elements were evenly distributed on the surface of SrCO_3_ crystals obtained in the microbial mineralization system, see Figure 6g Supporting Information Figure S4. Among them, the N elements in the SrCO_3_ crystals were derived from bacterial secretions. Moreover, thermal analysis (TA) data further verified the result that bacterial and nano-MMT took part in the crystallization process of SrCO_3_ crystals, see Supporting Information, Figure S5. In addition, see Supporting Information, Figure S6, shows that the morphology of the SrCO_3_ prepared by the chemical method was significantly different from the microbial method.

In addition, conductivity changes are used to further characterize the concentration of free ions in the solution. The decrease in the conductivity of the solution before and after the test reflects a process in which the positive and negative ions in the solution combine to form a non-conductive substance [30,31]. The conductivity of the mineralized solution in the presence of nano-MMT was significantly lower than that of the mineralized solution in the absence of nano-MMT. However, with the prolongation of the mineralization time, the conductivity showed a trend of increasing sharply first and then, more gradually, see Supporting Information, Figure S7. The main reason for the decrease in conductivity is attributed to the sufficient contact reaction between Sr^2+^ and CO_3_^2−^ in the mineralized solution. In addition, there is a strong interaction between the Sr^2+^ cation and the organic macromolecule, neutralizing the charged surface of the macromolecule [32]. Eventually, the molecules form agglomerates, or the metal chelate compound Sr^2+^ is formed to slow the change in the conductivity of the solution. However, the difference in the final conductivity values between the two systems was small, further indicating that nano-MMT was involved in the crystallization of SrCO_3_ crystals.

### 3.4. Mineralization Mechanism

Bacillus pasteuris decomposes urea as an energy source by secreted urease to produce CO_3_^2−^ in the microbial mineralization system. The CO_3_^2−^ formed by the hydrolysis of urea combined with the surrounding Sr^2+^ to form the amorphous strontium carbonate precursor, see Supporting Information, Figure S8. The extremely unstable amorphous strontium carbonate precursor rapidly transforms into SrCO_3_ crystals via dissolution and recrystallization. The spherical SrCO_3_ crystals gradually aggregated together via dissolution and recrystallization under the action of microbial secretion to form popcorn-like SrCO_3_ crystals. Because of its negative charge, Bacillus pasteuris itself acted as a nucleation site to attract strontium ions and the popcorn-like SrCO_3_ crystals are formed under the secretion of microorganisms during the dissolution and recrystallization [24].

**Scheme 1:**
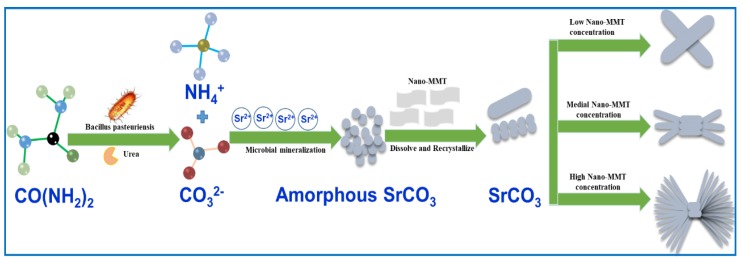
Formation of flower-like with superstructure SrCO_3_ in the presence of nano-MMT.

Based on the above experimental results, we have proposed the formation mechanism of the SrCO_3_ crystal with superstructures, as shown in Scheme 1. First, amorphous strontium carbonate precursor was formed at the initial stage of mineralization and then aggregated to form the original spherical SrCO_3_ crystals. With the mineralization time increasing, the nanostructured spherical morphology did not change significantly in the aggregated state and crystallization. Therefore, the ordered arrangement of spherical SrCO_3_ nanocrystals aggregated primary building blocks (rod-like SrCO_3_) formed via the van der Waals force and electrostatic interaction, and then gradually formed cross-shaped SrCO_3_ aggregates via the dissolution and recrystallization process under the attraction of nano-MMT [25,26]. With the increasing concentration of nano-MMT, a second nucleation process occurs on the as-prepared cross-shaped surface. Then, the primary building blocks form a flower-like SrCO_3_ crystal via a self-assembly process. This is consistent with the results proposed by Yu et al. that nano-blocky calcium carbonate forms a complex ellipsoidal calcium carbonate superstructure through both lattice geometric matching and the soft epitaxy effect [25]. These so-called intermediate crystallites interact and align in some form by dipole and electrostatic interactions, and then the intermediate crystallites were fused to each other to achieve the minimum total surface energy, and finally, a flower-like superstructure of SrCO_3_ crystals was produced [28].

### 3.5. Mineralization of Strontium Ions under Microbial Mineralization Systems

**Figure 7:**
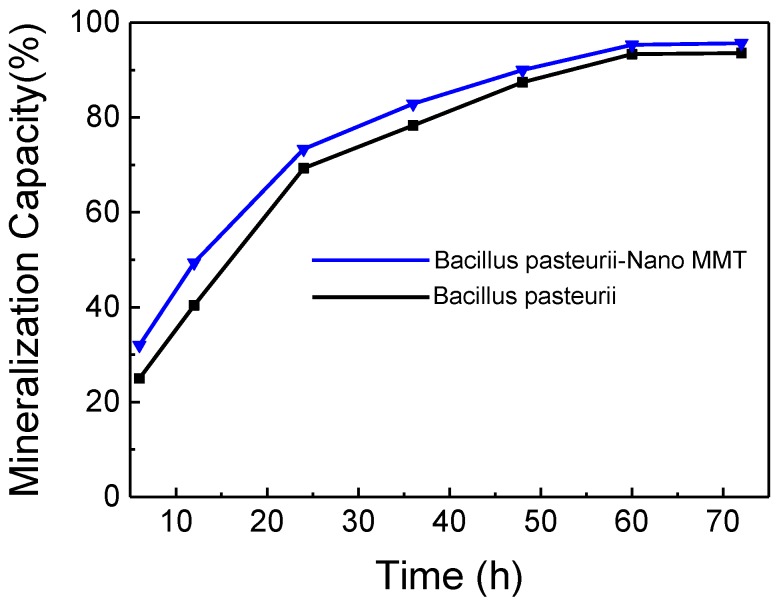
Mineralization efficiency of strontium ions in microbial mineralization systems.

Based on the previous research, we further investigated the mineralization efficiency of microbial synergistic nano-MMT to simulated radionuclide strontium. Figure 7 displayed the mineralization capacity of strontium ions by Bacillus pasteurii and Bacillus pasteurii-nano-MMT under different periods of time. With the extension of the mineralization time, the mineralization capacity of Bacillus pasteurii to strontium ions can reach more than 90%, regardless of the presence or absence of nano-MMT. However, the mineralization rate of Bacillus pasteurii to strontium ions was faster in the presence of nano-MMT, which may be attributed to the adsorption of strontium ions onto the surface of nano-MMT, thereby reducing the toxic effects of strontium ions on microorganisms [33].

In order to explore the effect of different pH values on the production of SrCO_3_, Figure S9, see Supporting Information, shows the effect of the pH value on the amount of SrCO_3_ produced by microorganisms. It can be seen from Figure S9 that a different pH value has a great influence on the production of SrCO_3_, and the yield of SrCO_3_ reaches the maximum at pH = 8. Interestingly, the yield of SrCO_3_ in the presence of nano-MMT was significantly higher than that of the control group, which may be attributed to the fact that nano-MMT can buffer the pH value of the mineralization system. The above results also demonstrate that pH value has a greater impact on biomineralization.

## 4. Conclusions

In summary, nano-MMT was used as a crystal growth regulator to prepare SrCO_3_ with a complex flower-like superstructure in a microbial mineralization system. The effects of nano-MMT concentration and mineralization time on the phase transition and morphology of SrCO_3_ were systematically studied. An emerging self-assembly process and crystal polymerization mechanism have been proposed by forming complex flower-like SrCO_3_ superstructures in high concentrations of nano-MMT. These results indicated that unique bionic synthesis strategies provide a useful route for the production of inorganic or inorganic/organic composites with distinctive morphologies and unique structures in microbial mineralization systems but also provide new ideas for the treatment of radionuclides.

## Supplementary Materials

The following are available online at https://www.mdpi.com/1996-1944/12/9/1392/s1, Figure S1: (**a**) Bacillus pasteurii colony, (**b**) SEM of bacillus pasteurii mycelium, Figure S2: (**a**) TEM images of nano-MMT, where the inset shows pictures of nano-MMT suspension, (**b**) XRD patterns of nano-MMT, Figure S3: (**a**) XRD patterns and (**b**) FT-IR spectrum of strontium carbonate obtained using varying amounts of nano-MMT, Figure S4: EDS spectrum of strontium carbonate, Figure S5: TGA spectra of mineralized samples in the absence of additives and the presence of nano-MMT, respectively, Figure S6: Typical SEM images of SrCO_3_ were obtained by chemical method. (**a**) SrCO_3_ were obtained in pure water, (**b**) mineralized samples were obtained in the presence of nano-MMT, Figure S7: SEM of amorphous SrCO_3_ precursor in the early stage of microbial mineralization, Figure S8: Relationship of conductivity with time in different mineralized solutions, Figure S9: The effect of pH values on the yield of SrCO_3_.
