# Supplementary material for: Nano-Montmorillonite Regulated Crystallization of Hierarchical Strontium Carbonate in a Microbial Mineralization System
Source: Materials (Basel). 2019 Apr 29;12(9):1392. doi: 10.3390/ma12091392 (PMC6539429; doi:10.3390/ma12091392)
Supplement: Supplementary file 1 [file materials-12-01392-s001.pdf]

Nano-montmorillonite Regulated Crystallization of Hierarchical Strontium Carbonate in a Microbial Mineralization System

Kui Zheng [1,#] Tao Chen [2,3,#], Jian Zhang [2,3,#], Xiuquan Tian [2], Huilin Ge [2],Tiantao Qiao [2], Jia Lei [2,3], Xianyan Li [2], Tao Duan [2,3] and Wenkun Zhu [2,3,*]

[1] Analytical and Testing Center, Southwest University of Science and Technology, Mianyang 621010, China; zhengkui@swust.edu.cn

[2] State Key Laboratory of Environmentally Friendly Energy Materials, Southwest University of Science and Technology, Mianyang 621010, China; chentao314710@163.com (T.C.); z2358727542@163.com (J.Z.); tian1993tian1993@163.com (X.T.); gehuilin1998@163.com (H.G.); 1805338097@qq.com (T.Q.); cutiancheng@163.com (J.L.); lxying@swust.edu.cn (X.L.); duant@ustc.edu.cn (T.D.)

[3] Nuclear Waste and Environmental Safety Key Laboratory of Defense, National Collaborative Innovation Center for Nuclear Waste and Environmental Safety, Southwest University of Science and Technology, Mianyang 621010, China

* Correspondence: zhuwenkun@swust.edu.cn; Tel.: +86-816-608-9883

[#] These authors contributed equally.

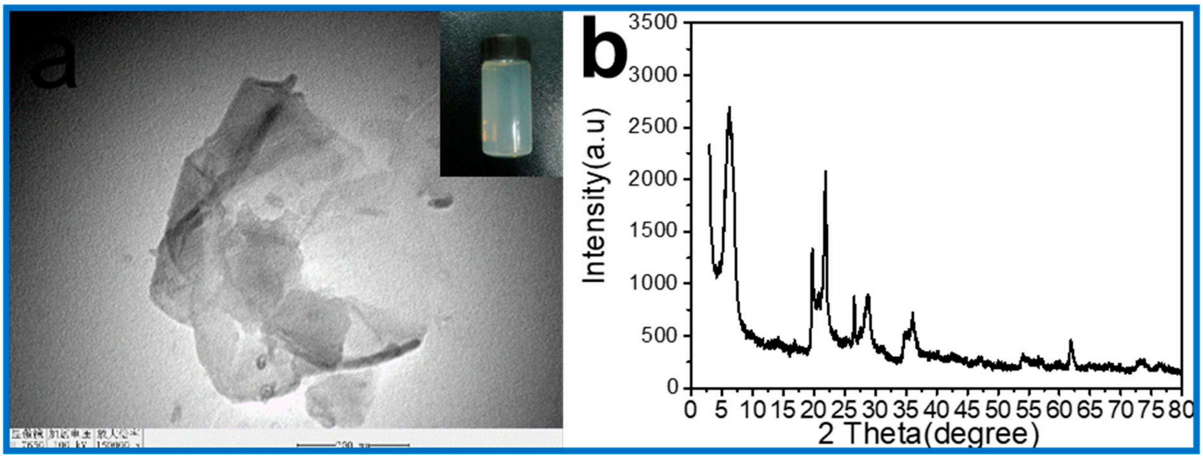

Figure S1. (**a**) TEM images of nano-MMT, where the inset shows pictures of nano-MMT suspension, (**b**) XRD patterns of nano-MMT.

The colony of Bacillus pasteurii on LB agar medium is characterized by smooth colonies, homogeneous edges, clear, elevated, viscous, see Figure S2a. Bacillus pasteurii is a spherical bacterium with a smooth surface and a size of 0.8–1.5 μm, see Figure S2b. The morphology of Bacillus pasteurii is distinctly different from that of calcite rods.

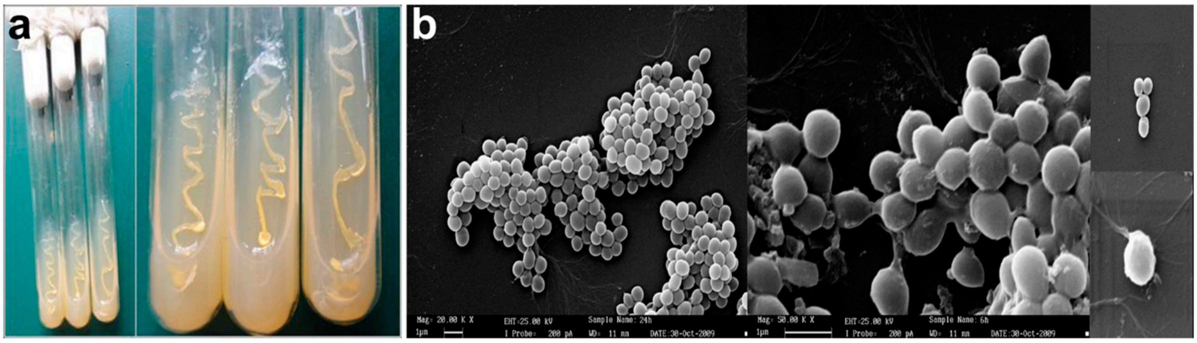

Figure S2. (**a**) Bacillus pasteurii colony, (**b**) SEM of bacillus pasteurii mycelium.

In the process of microbial mineralization of calcium carbonate, microorganisms play two core roles: One is to provide urease for urea hydrolysis, and the other is to provide crystal nuclei for the formation of calcium carbonate crystals [1,2,4,5]

$$NH_2\text{-}CO\text{-}NH_2 + 3H_2O \rightarrow 2NH_4^+ + 2OH^- + CO_2 \quad (1)$$

$$Sr^{2+} + Cell \rightarrow Cell\text{-}Sr^{2+} \quad (2)$$

$$Cl^- + HCO^{3-} + NH_3 \rightarrow NH_4Cl + CO_3^{2-} \quad (3)$$

$$Cell\text{-}Sr^{2+} + CO_3^{2-} \rightarrow Cell\text{-}SrCO_3 \downarrow \quad (4)$$

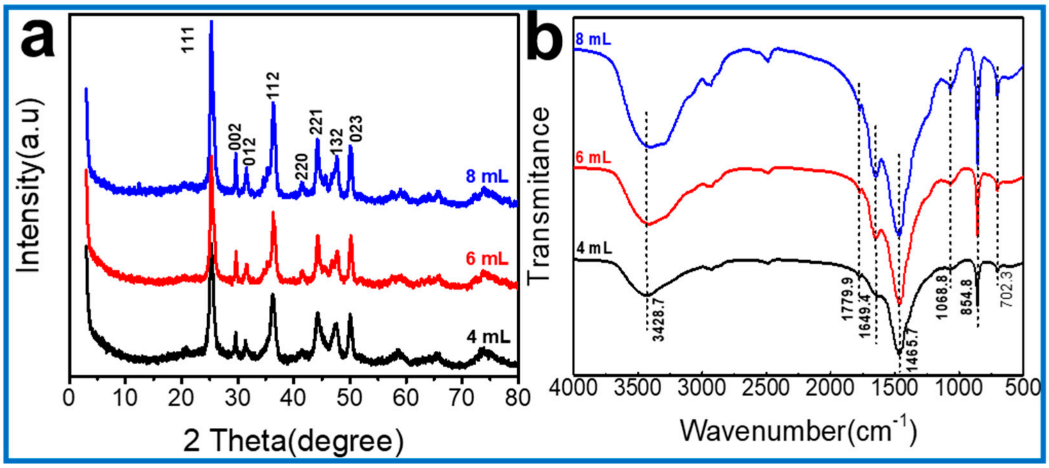

Figure S3. (**a**) XRD patterns and (**b**) FT-IR spectrum of strontium carbonate obtained using varying amounts of nano-MMT.

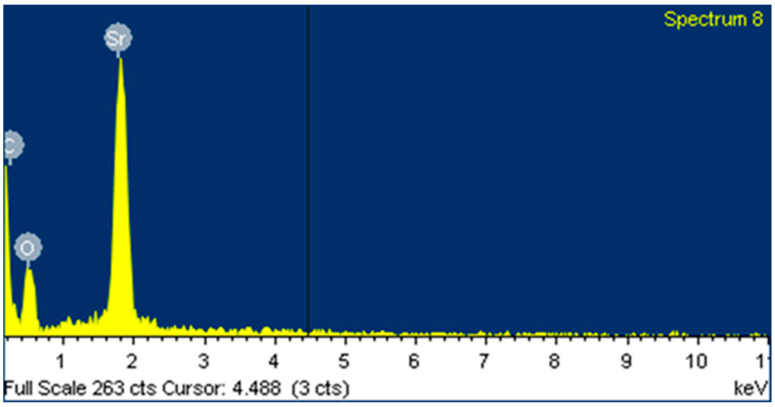

Figure S4. EDS spectrum of strontium carbonate.

Figure S5 shows the TGA data of strontium carbonate. The entire decomposition process was accompanied by three weight loss stages [3,6]. Weight lost slowly should be caused by the loss of water under 290 °C. Notable weight loss appeared in the curve during 313.5–776.3 °C, which should be caused by thermal decomposition of bacteria metabolite in the mineralized samples. The mineralized samples in the presence of nano-MMT have a weight loss rate of 5.0%, the mineralized samples in the absence of nano-MMT have a weight loss rate of 3.9%. Evidently, the weight loss rate of the mineralized samples in the presence of nano-MMT was 1.1% greater than the weight loss rate of the mineralized samples in the absence of nano-MMT, which should be attributed to the pyrolysis of nano-MMT in mineralized samples. Large thermal decomposition occurs at 776.3–985.6 °C, which can be attributed to the decomposition of strontium carbonate. From the analysis above, it is safe to conclude that the nano-MMT was involved in the crystallization process of strontium carbonate.

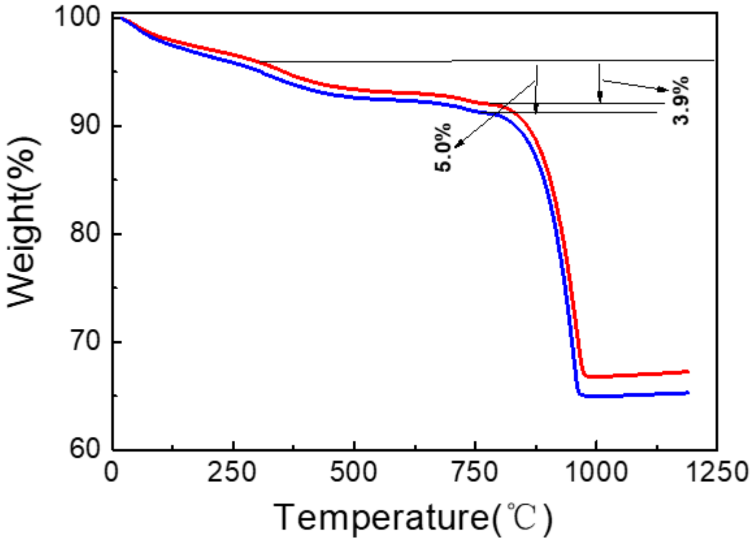

Figure S5. TGA spectra of mineralized samples in the absence of additives and the presence of nano-MMT, respectively.

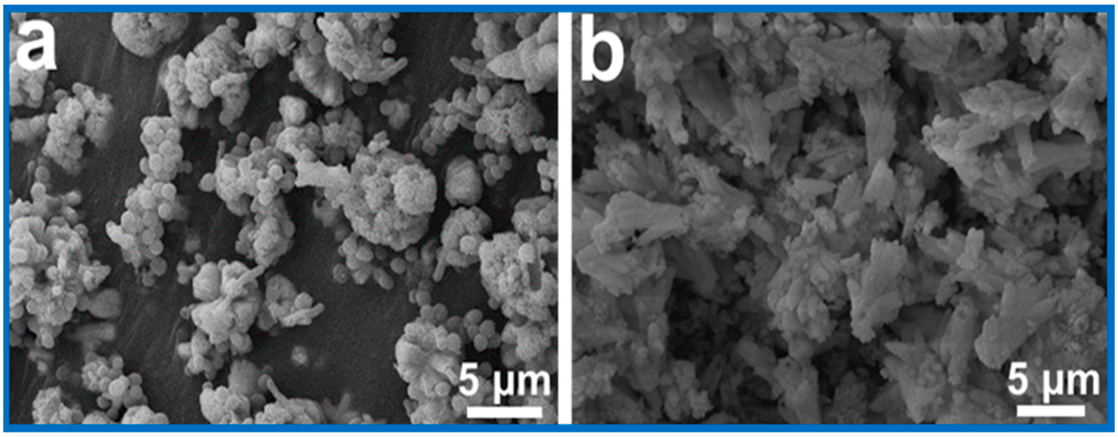

Figure S6. Typical SEM images of $SrCO_3$ were obtained by chemical method. (**a**) $SrCO_3$ were obtained in the water, (**b**) mineralized samples were obtained in the presence of Nano-MMT.

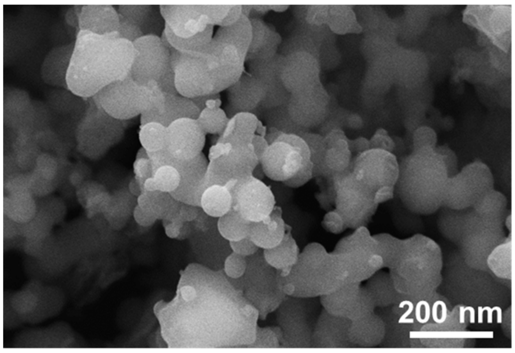

Figure S7. SEM of amorphous $SrCO_3$ precursor in the early stage of microbial mineralization.

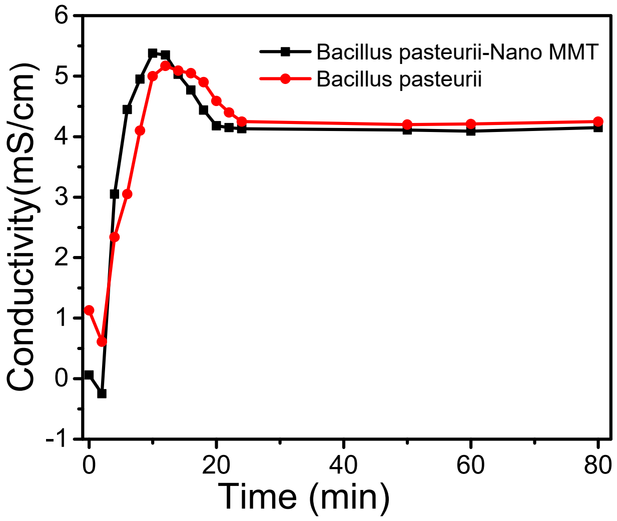

Figure S8. Relationship of conductivity with time in different mineralized solutions.

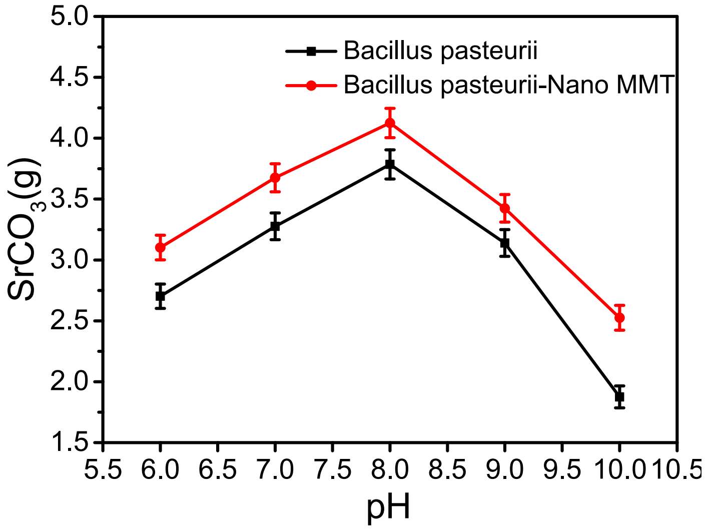

Figure S9. The effect of pH values on the yield of $SrCO_3$.

References

1. Dejong, J.T.; Fritzges, M.B.; Nüsslein, K. Microbially Induced Cementation to Control Sand Response to Undrained Shear. *J. Geotech. Geoenviron. Eng.* **2006**, *132*, 1381–1392.
2. Muynck, W.D.; Belie, N.D.; Verstraete, W.; Jonkers, H.M.; Loosdrecht, M.C.M.V. Microbial carbonate precipitation in construction materials: A review. *Ecolog. Eng*. **2010**, *36*, 118–136.
3. Chen, T.; Li, J.; Shi, P.; Li, Y.; Lei, J.; Zhou, J.; Hu, Z.; Duan, T.; Tang, Y.; Zhu, W. Effects of Montmorillonite on the Mineralization and Cementing Properties of Microbiologically Induced Calcium Carbonate. *Adv. Mater. Sci. Eng.* **2017**, *2017*, 1–13.
4. Chen, T.; Shi, P.; Li, Y.; Zhang, J.; Duan, T.; Yu, Y.; Zhou, J.; Zhu, W. Crystallization of calcium carbonate mineral with hierarchical structures regulated by silk fibroin in microbial mineralization system. *J. Cryst. Growth*, **2018**, *493*, 51–57.
5. Chen, T.; Shi, P.H.; Li, Y.; Duan, T.; Yu, Y.; Li, X.; Zhu, W. Biomineralization of Varied Calcium Carbonate Crystal by Synergistic Effect of Silk Fibroin/Magnesium ions in a Microbial System. *Crystengcomm* **2018**, *20*, 2366–2373.
6. Liu, L.; Zhang, X.; Liu, X.; Liu, J.; Lu, G.; Kaplan, D.L.; Zhu, H.; Lu, Q. Biomineralization of stable and monodisperse vaterite microspheres using silk nanoparticles. *Acs Appl. Mater. Interfaces* **2015**, *7*, 1735–1745.

.
